# Dose-dependent effects of apilarnil on muscle damage and endurance performance during exhaustive swimming exercise in rats: translational ınsights for sports physiology

**DOI:** 10.1007/s00421-026-06205-w

**Published:** 2026-03-31

**Authors:** Fatih Çakar, Halil Şimşek

**Affiliations:** Vocational School of Health Services, Bingöl University, Bingöl, Turkey

**Keywords:** Apilarnil, Oxidative stress, Inflammation, Endurance performance, Skeletal muscle, Cardiac muscle, Nutraceuticals

## Abstract

**Purpose:**

Intensive exercise is known to induce muscle damage accompanied by excessive oxidative stress and inflammatory responses, which may compromise performance and recovery. Apilarnil, a nutrient-rich bee product containing bioactive phenolic compounds and flavonoids, has been proposed to exert antioxidant and anti-inflammatory effects; however, its role in exercise-induced physiological stress remains insufficiently characterized.

**Methods:**

Male Wistar rats were allocated into five groups: Control (CN), Without Exercise and Apilarnil (WEAP), Exercise (EX), Exercise + Apilarnil 0.2 g^.^kg^−1^ (EX + AP1), and Exercise + Apilarnil 0.4 g^.^kg^−1^ (EX + AP2). Exercised rats performed daily swimming (30 min/day) for 14 days, while apilarnil (0.2 or 0.4 g·kg^−1^) or vehicle was administered once daily throughout the 14-day protocol. An exhaustive swimming test was conducted at the end of the 14-day period, after which serum muscle damage markers, lipid peroxidation, pro-inflammatory cytokines, and antioxidant enzyme activities were assessed in skeletal and cardiac muscle tissues.

**Results:**

Compared with WEAP, exhaustive swimming increased muscle damage markers, lipid peroxidation, and inflammatory cytokine levels. Compared with EX, apilarnil supplementation attenuated oxidative and inflammatory responses and enhanced endogenous antioxidant enzyme activities. Notably, the lower apilarnil dose resulted in the greatest improvement in swimming performance, whereas performance benefits were attenuated at the higher dose despite favorable biochemical profiles.

**Conclusion:**

Apilarnil supplementation modulated exercise-induced oxidative stress and inflammatory responses in skeletal and cardiac muscle tissues, with dose-dependent effects observed primarily in biochemical markers. In contrast, endurance performance follows a non-linear pattern, peaking at the lower dose and diminishing at the higher dose, underscoring the need to identify an optimal ergogenic dose.

**Graphical abstract:**

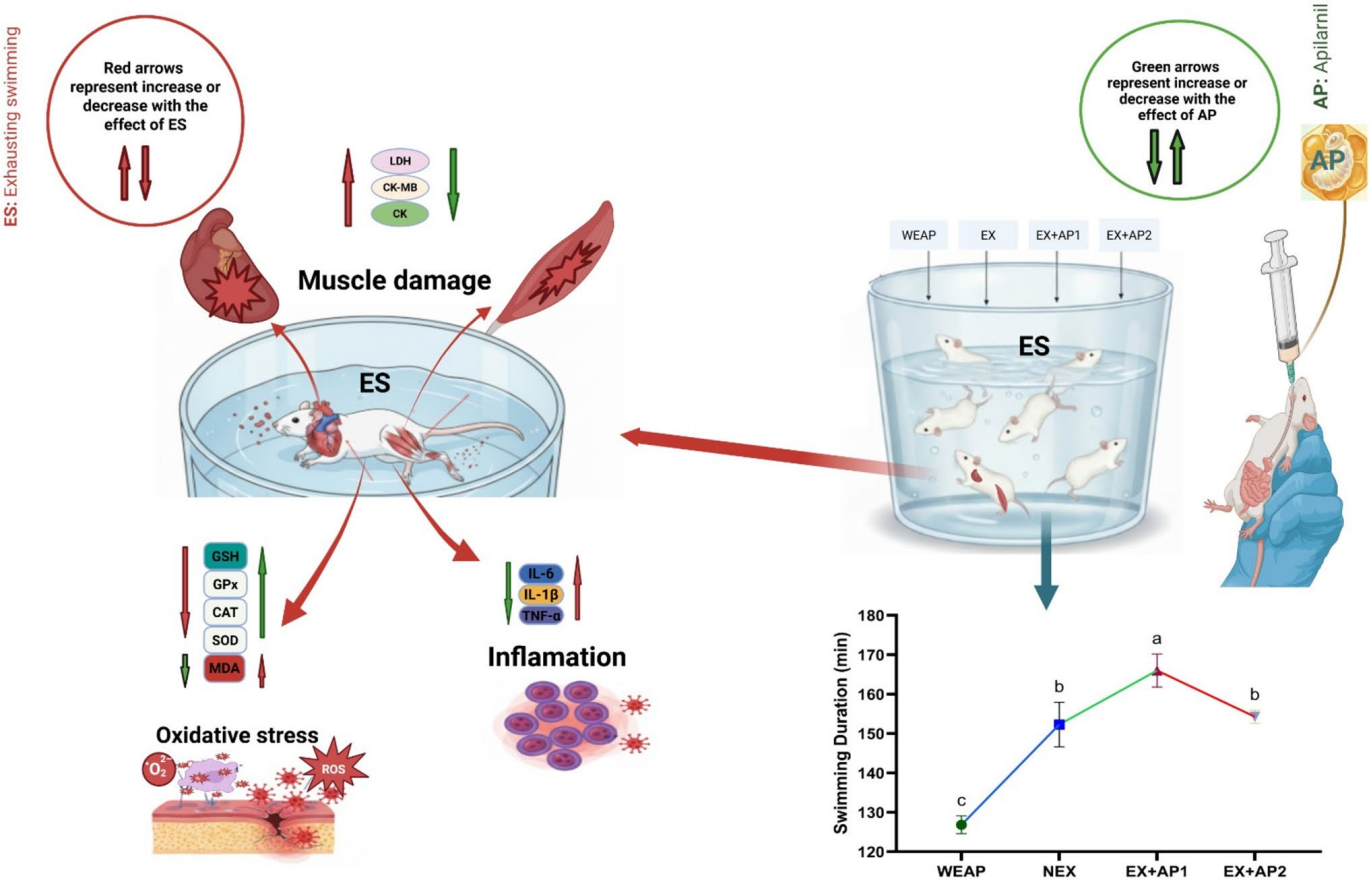

## Introduction

Intensive and exhaustive exercise places substantial mechanical and metabolic demands on the organism, frequently leading to muscle damage accompanied by oxidative stress and inflammatory responses (Peake et al. 2017; Thirupathi et al. 2021b). During high-intensity physical activity, increased oxygen consumption promotes excessive reactive oxygen species (ROS) production, which can disrupt redox homeostasis and trigger lipid peroxidation, cellular damage, and inflammatory signaling pathways (Wang et al. 2021; Thirupathi et al. 2021b; Duranti 2023). Although these responses contribute to physiological adaptation when tightly regulated, excessive or prolonged oxidative and inflammatory stress may impair muscle function and recovery processes; however, the relationship between biochemical stress markers and functional performance outcomes is complex and not necessarily linear (Peake et al. 2017; Margaritelis et al. 2020).

Exercise-induced oxidative stress is closely linked to inflammatory responses mediated by pro-inflammatory cytokines such as tumor necrosis factor-α (TNF-α), interleukin-1β (IL-1β), and interleukin-6 (IL-6) (Peake et al. 2017; Thirupathi et al. 2021a). Experimental studies in rats have demonstrated that acute exhaustive exercise markedly elevates oxidative stress markers and inflammatory mediators, particularly in unadapted tissues (Kabakçı and Çınar 2021; Thirupathi et al. 2021b). While skeletal muscle has been the primary focus of most investigations, emerging evidence suggests that cardiac muscle is also vulnerable to exercise-induced redox imbalance and inflammation, highlighting the systemic nature of exercise-related physiological stress (Margaritelis et al. 2020).

Because oxidative stress and inflammatory responses are involved in exercise adaptation and recovery processes, nutritional strategies aimed at modulating these responses have gained increasing attention in exercise physiology research (Powers et al. 2016; Yang et al. 2020; Duranti 2023). In particular, natural products rich in bioactive compounds are considered promising candidates due to their potential to provide balanced antioxidant and anti-inflammatory modulation without fully suppressing exercise-induced adaptive signaling (Thirupathi et al. 2021b; Taherkhani et al. 2021; Wang et al. 2024). Within this context, bee products have attracted growing scientific interest owing to their diverse biological activities and favorable safety profiles (Denisow and Denisow‐Pietrzyk 2016; El-Seedi et al. 2022).

Apilarnil is a lesser-known bee product derived from male honeybee (*Apis mellifera*) larvae and is characterized by a complex composition including proteins, essential amino acids, lipids, vitamins, and phenolic compounds (Silici 2019; Mărgăoan et al. 2019; Babacan and Ayar 2024). Previous studies have demonstrated that apilarnil exhibits antioxidant and anti-inflammatory properties and may exert protective effects in various experimental models, including liver, kidney, and nervous system injury (Hamamci et al. 2020; Doğanyiğit et al. 2020; Inandiklioglu et al. 2021). In addition, comparative analyses have highlighted the rich bioactive profile of apilarnil relative to other bee-derived products, supporting its potential functional relevance (Mărgăoan et al. 2017; İvgin Tunca and Arslan 2024).

Despite these findings, evidence regarding the effects of apilarnil in the context of exercise-induced physiological stress remains limited, particularly with respect to the interaction between biochemical responses and functional performance outcomes. To date, only a small number of studies have examined the impact of apilarnil on exercise-related outcomes, and these have primarily focused on organ-specific injury rather than integrated assessments of muscle oxidative stress, inflammation, and performance (Çakar et al. 2025). Moreover, potential dose-dependent effects of apilarnil supplementation during intensive exercise have not been systematically investigated, and its influence on both skeletal and cardiac muscle responses remains unclear.

Therefore, the present study aimed to investigate the effects of apilarnil supplementation on exercise-induced muscle damage, oxidative stress, inflammatory responses, and swimming performance in a rat model of exhaustive exercise. By simultaneously evaluating skeletal and cardiac muscle tissues and incorporating functional performance outcomes, this study seeks to provide translational insights into the potential role of apilarnil as a natural ergogenic support under conditions of intensive physical stress.

### Study hypotheses

- H1: Apilarnil supplementation modulates exercise-induced redox imbalance and inflammatory responses in skeletal and cardiac muscle tissues under exhaustive exercise conditions.
- H2: Apilarnil supplementation improves endurance performance, as measured by swimming time to exhaustion, in rats exposed to intensive exercise.
- H3: The effects of apilarnil supplementation on oxidative stress markers, inflammatory mediators, antioxidant enzyme activities, and endurance performance are dose-dependent.

## Materials and methods

### Experimental animals and ethical approval

A total of 35 adult male Wistar albino rats (250–300 g) were used in this study. Prior to the experiment, the animals were housed under standard laboratory conditions (22 ± 2 °C temperature, 50–60% humidity, and a 12:12 h light/dark cycle) and were provided ad libitum access to standard rat chow and water. The experimental protocol was approved by the Animal Experiments Local Ethics Committee of a state university (Approval No: E-856802-020-200009353/2026-revised), and all experimental procedures were conducted in accordance with the ARRIVE guidelines. Only male rats were used in order to minimize potential variability associated with hormonal fluctuations during the estrous cycle, which may influence exercise performance and biochemical responses.

### Procurement and preparation of apilarnil

The apilarnil (AP) used in this study was obtained from Harşena Bee Products (Amasya, Türkiye). For the 0.2 g^.^kg^−1^ and 0.4 g^.^kg^−1^ doses, apilarnil was dissolved and homogenized in distilled water (Doğanyiğit et al. 2020). All animals received a single daily dose of AP administered via oral gavage in a total volume of 0.5 mL.

### Bioactive composition of apilarnil used in the study

According to the LC–MS/MS analysis of the AP preparation used in this study, the extract was found to be rich in bioactive phenolic acids and flavonoids, including quinic acid (29.863 mg^.^kg^−1^ extract), *p*-coumaric acid (6.080 mg^.^kg^−1^ extract), caffeic acid (3.804 mg^.^kg^−1^ extract), protocatechuic acid (4.404 mg^.^kg^−1^ extract), quercetin (3.023 mg^.^kg^−1^ extract), kaempferol (2.125 mg^.^kg^−1^ extract), and acacetin (2.189 mg^.^kg^−1^ extract) (Çakar and Şimşek 2025).

### Experimental design and randomization

Group size (n = 7 rats/group) was selected a priori in line with comparable rat exhaustive-swimming exercise studies evaluating endurance performance together with exercise-related oxidative stress and inflammatory biomarkers in both skeletal and cardiac muscle. For transparency, the primary outcome variables used for the power assessment were defined as (i) endurance performance (time to exhaustion during the exhaustive swimming test) and (ii) lipid peroxidation measured in biceps femoris (skeletal muscle) and left ventricle (cardiac muscle), as these endpoints directly reflect the main functional and mechanistic aims of the study. A post hoc power analysis was conducted using G*Power 3 for one-way ANOVA (five groups; α = 0.05) based on the observed effect sizes for these primary endpoints, indicating an achieved power > 80% (Faul et al. 2007).

### Experimental groups

The animals were divided into the following experimental groups:

Control (CN): No exercise or supplementation was applied.

Without Exercise and Apilarnil (WEAP): Rats received 0.5 mL of physiological saline orally for 14 days without exercise or apilarnil supplementation.

Exercise (EX): Rats were subjected to swimming exercise for 30 min per day for 14 days and received 0.5 mL of physiological saline orally.

EX + AP1: Rats were subjected to swimming exercise for 30 min per day for 14 days and received apilarnil at a dose of 0.2 g^.^kg^−1^ orally.

EX + AP2: Rats were subjected to swimming exercise for 30 min per day for 14 days and received apilarnil at a dose of 0.4 g^.^kg^−1^ orally.

Apilarnil doses were selected based on previously reported antioxidant and adaptogenic effects in the literature (Doğanyiğit et al. 2020; Inandiklioglu et al. 2021).

### Swimming exercise protocol and performance assessment

Animals in the exercise groups underwent a one-week water acclimation period prior to the experimental protocol. Rats in the WEAP group also underwent the same one-week water acclimation procedure to ensure comparable water exposure conditions, although they did not participate in the training protocol. All swimming exercise sessions and performance assessments were conducted during the light phase of the light–dark cycle (between 09:00 and 12:00) to minimize circadian variability. Swimming exercise and performance testing were performed in a Morris-type cylindrical swimming tank (130 cm in diameter and 50 cm in depth), with the water temperature maintained at 31.0 ± 1.0 °C throughout the experiment. Following completion of the 14-day swimming protocol, all experimental groups except the control group were subjected to an exhaustive swimming test. Each rat was individually placed in the tank, and the time to exhaustion was recorded in seconds. Exhaustion was defined as the loss of coordinated swimming movements and the inability to return to the water surface for at least 10 s despite gentle stimulation. This behavioral criterion is widely accepted as an indicator of maximal exercise tolerance in rat swimming models, and exercise intensity was determined based on established behavioral exhaustion criteria (Powers et al. 2016; Veskoukis 2018). Swimming duration was used as an indicator of endurance performance. Immediately after the exhaustive swimming test, animals were anesthetized with xylazine (10 mg^.^kg^−1^; Rompun®, Bayer, Germany) and ketamine (100 mg^.^kg^−1^; Ketalar®, Pfizer, Türkiye).

### Sample collection and storage

At the end of the experimental protocol, blood samples were collected from the abdominal vein using a sterile syringe and transferred into anticoagulant-free tubes. The blood was allowed to clot at room temperature for approximately 30 min before centrifugation. Samples were then centrifuged at 3000 rpm for 10 min at 4 °C to obtain serum, and serum aliquots were stored at −80 °C until analysis (Yang et al. 2020). Following blood collection, animals were euthanized by decapitation. For tissue analyses, the biceps femoris muscle (skeletal muscle) and the left ventricle (cardiac muscle) were rapidly excised, briefly rinsed in ice-cold saline to remove residual blood, blotted dry, snap-frozen in liquid nitrogen, and stored at −80 °C until further processing.

### Parameters assessed

The following parameters were evaluated in this study:

Serum muscle damage markers: Creatine kinase (CK), creatine kinase-MB (CK-MB), and lactate dehydrogenase (LDH).

Oxidative stress and antioxidant parameters in skeletal and cardiac muscle tissues: Activities of superoxide dismutase (SOD), catalase (CAT), and glutathione peroxidase (GSH-Px), as well as levels of malondialdehyde (MDA) and reduced glutathione (GSH).

Inflammatory markers in skeletal and cardiac muscle tissues: Tumor necrosis factor-α (TNF-α), interleukin-1β (IL-1β), and interleukin-6 (IL-6).

Performance assessment: Time to exhaustion during swimming in the Morris-type tank.

### Tissue preparation, homogenization, and protein determination

Cardiac and skeletal muscle tissues were processed under cold-chain conditions. The biceps femoris (skeletal muscle) and left ventricle (cardiac muscle) samples were homogenized in ice-cold phosphate-buffered saline (PBS; 50 mM, pH 7.4) at a tissue-to-buffer ratio of 1:10 (w/v) using a mechanical homogenizer. The homogenates were centrifuged at 10,000 × g for 15 min at 4 °C, and the resulting supernatants were collected for subsequent biochemical analyses.

Protein concentrations in the tissue supernatants were determined using the Bradford method with a commercially available protein assay kit, using bovine serum albumin (BSA) as the standard. All biochemical measurements were normalized to milligrams of protein.

### Oxidative stress and antioxidant analyses

Malondialdehyde (MDA; lipid peroxidation): MDA levels were determined according to the method of Placer et al. (1966). Briefly, samples were reacted with thiobarbituric acid (TBA) followed by heating and cooling steps, and the absorbance was measured at 532 nm.

Reduced glutathione (GSH): Samples were precipitated with trichloroacetic acid (TCA), and Tris buffer and 5,5′-dithiobis-(2-nitrobenzoic acid) (DTNB) were added to the supernatant. Absorbance was measured at 412 nm.

Glutathione peroxidase (GSH-Px): GSH-Px activity was determined according to the method of Matkovics (1988). Control and sample mixtures were prepared and incubated, after which the reaction was terminated by the addition of TCA. Absorbance was measured at 412 nm, and results were expressed as IU/mg protein.

Catalase (CAT): CAT activity was measured according to the method of Aebi by monitoring the decomposition of hydrogen peroxide (H₂O₂) following the addition of tissue supernatant (Aebi 1984).

Superoxide dismutase (SOD): SOD activity was determined according to the method of Sun et al. (1988). using the xanthine/xanthine oxidase system and nitroblue tetrazolium (NBT), with absorbance measured at 560 nm.

### Measurement of proinflammatory cytokines by ELISA

The levels of tumor necrosis factor-α (TNF-α), interleukin-1β (IL-1β), and interleukin-6 (IL-6) in tissue supernatants were quantified using commercially available sandwich ELISA kits (Shanghai Korain Biotech Co., Ltd., Jiaxing, China). Samples were incubated at 37 °C, followed by sequential washing steps and the addition of conjugated antibodies and substrate solution. The reaction was terminated with a stop solution, and absorbance was measured at 450 nm using a microplate reader (Multiskan GO, Thermo Scientific, Vantaa, Finland). Cytokine concentrations were calculated based on standard calibration curves.

### Serum biochemical analyses

Serum levels of creatine kinase (CK), creatine kinase-MB (CK-MB), and lactate dehydrogenase (LDH) were analyzed in a certified biochemistry laboratory through outsourced analytical services using standard automated methods.

### Statistical analysis

Statistical analyses were performed using GraphPad Prism version 10.3.1 (GraphPad Software, San Diego, CA, USA). The normality of data distribution was assessed using the Shapiro–Wilk test, and homogeneity of variances was evaluated using Levene’s test. Intergroup comparisons were conducted using one-way analysis of variance (ANOVA), followed by Bonferroni post hoc tests for multiple comparisons. Results are presented as mean ± standard error of the mean (SEM), and a *p* value < 0.05 was considered statistically significant.

## Results

### Inflammatory markers in skeletal and cardiac muscle tissues

**Fig.1:**
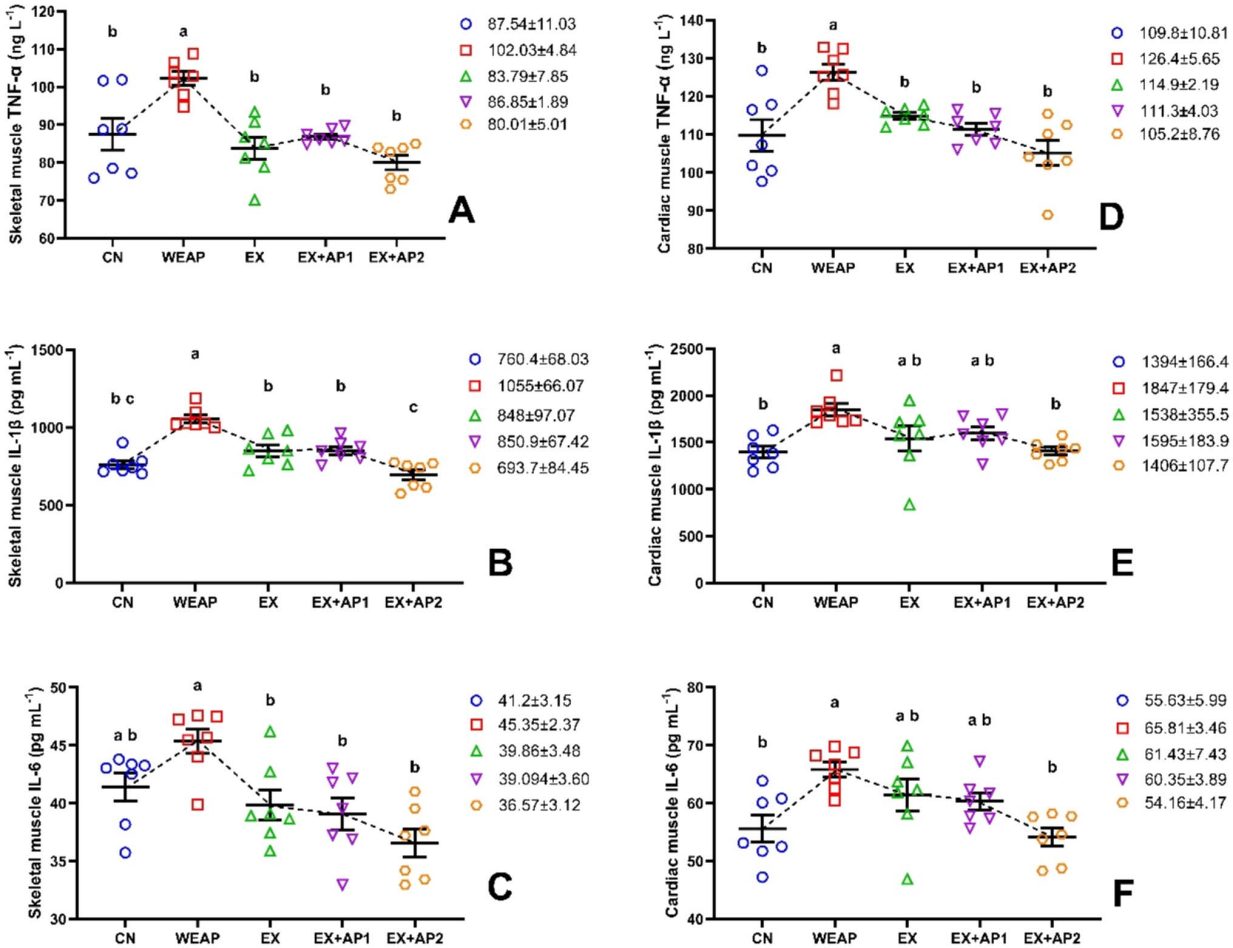
Pro-inflammatory cytokine levels in skeletal and cardiac muscle tissues.Pro-inflammatory cytokines were quantified by ELISA in skeletal muscle (biceps femoris) and cardiac muscle (left ventricle). **A-C** Skeletal muscle: TNF-α, IL-1β, and IL-6. **D-F** Cardiac muscle (left ventricle): TNF-α, IL-1β, and IL-6. Data are presented as mean ± SEM. One-way ANOVA followed by Bonferroni post hoc test; bars not sharing a common letter differ significantly (p < 0.05). *CN* Control, *WEAP* Without exercise and apilarnil, EX Exercise, *EX* + *AP1* Exercise + apilarnil (0.2 g^.^kg^−1^), *EX* + *AP2* Exercise + apilarnil (0.4 g^.^kg^−1^)

Compared with CN, the WEAP group exhibited significantly higher TNF-α and IL-1β levels in both skeletal and cardiac muscle tissues (p < 0.05) (Fig. 1A–C, D-F). Cardiac IL-6 was also significantly elevated in WEAP compared with CN (p < 0.05), whereas skeletal muscle IL-6 did not differ significantly between these groups (p > 0.05).

Relative to WEAP, the 14-day training protocol (EX) significantly reduced TNF-α, IL-1β, and IL-6 levels in skeletal muscle (p < 0.05). In cardiac muscle, training significantly reduced TNF-α compared with WEAP (p < 0.05), while IL-1β and IL-6 remained statistically unchanged (p > 0.05) (Fig. 1D–F).

Direct comparison with EX showed that EX + AP1 did not produce additional significant changes in cytokine levels in either skeletal or cardiac muscle (p > 0.05). In contrast, EX + AP2 resulted in a further reduction in skeletal muscle IL-1β compared with EX (p < 0.05), whereas skeletal TNF-α and IL-6, as well as all cardiac cytokines, remained comparable to EX (p > 0.05) (Fig. 1A–F).

### Serum muscle damage markers

Compared with CN, the WEAP group exhibited significantly higher serum CK, CK-MB, and LDH levels (p < 0.05), consistent with an acute muscle damage response following the exhaustive swimming test.

Relative to WEAP, the 14-day training (EX) showed significantly lower CK, CK-MB, and LDH levels (p < 0.05), suggesting a training-related attenuation of muscle damage markers after the exhaustive test.

**Fig. 2:**
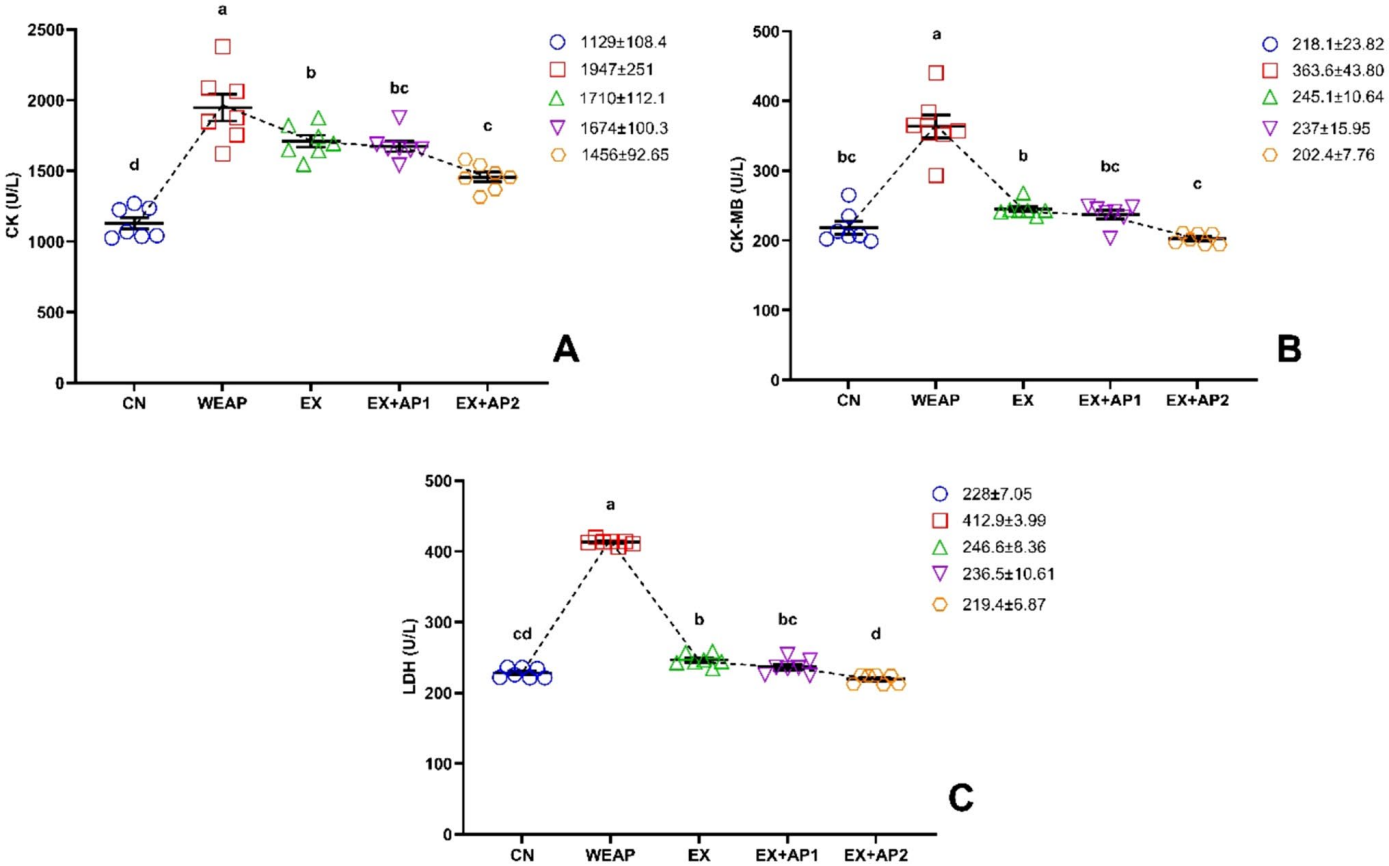
Serum muscle damage markers. **A** Creatine kinase (CK), **B** creatine kinase-MB (CK-MB), and **C** lactate dehydrogenase (LDH). Data are presented as mean ± SEM. One-way ANOVA followed by Bonferroni post hoc test; bars not sharing a common letter differ significantly (p < 0.05). *CN* Control, *WEAP* Without exercise and apilarnil, *EX* Exercise, *EX* + *AP1* Exercise + apilarnil (0.2 g^.^kg^−1^), *EX* + *AP2* Exercise + apilarnil (0.4 g^.^kg^−1^)

When compared directly with EX, EX + AP1 did not produce additional significant changes in these serum damage markers (p > 0.05). In contrast, EX + AP2 showed significantly lower CK, CK-MB, and LDH levels compared with EX (p < 0.05), suggesting an additional protective effect of the higher apilarnil dose beyond training alone (Fig. 2A–C).

### Oxidative stress and antioxidant parameters in skeletal muscle

Relative to CN, the WEAP group showed significantly lower antioxidant enzyme activities (SOD, CAT, and GSH-Px) (p < 0.05), accompanied by significantly higher MDA levels (p < 0.05), indicating an oxidative stress response following the exhaustive exercise test. GSH levels did not differ significantly between WEAP and CN (p > 0.05).

Compared with WEAP, the 14-day swimming training protocol (EX) resulted in significantly higher SOD, CAT, and GSH-Px activities (p < 0.05). MDA levels were significantly lower in EX than WEAP (p < 0.05), whereas GSH remained statistically unchanged (p > 0.05).

**Fig. 3:**
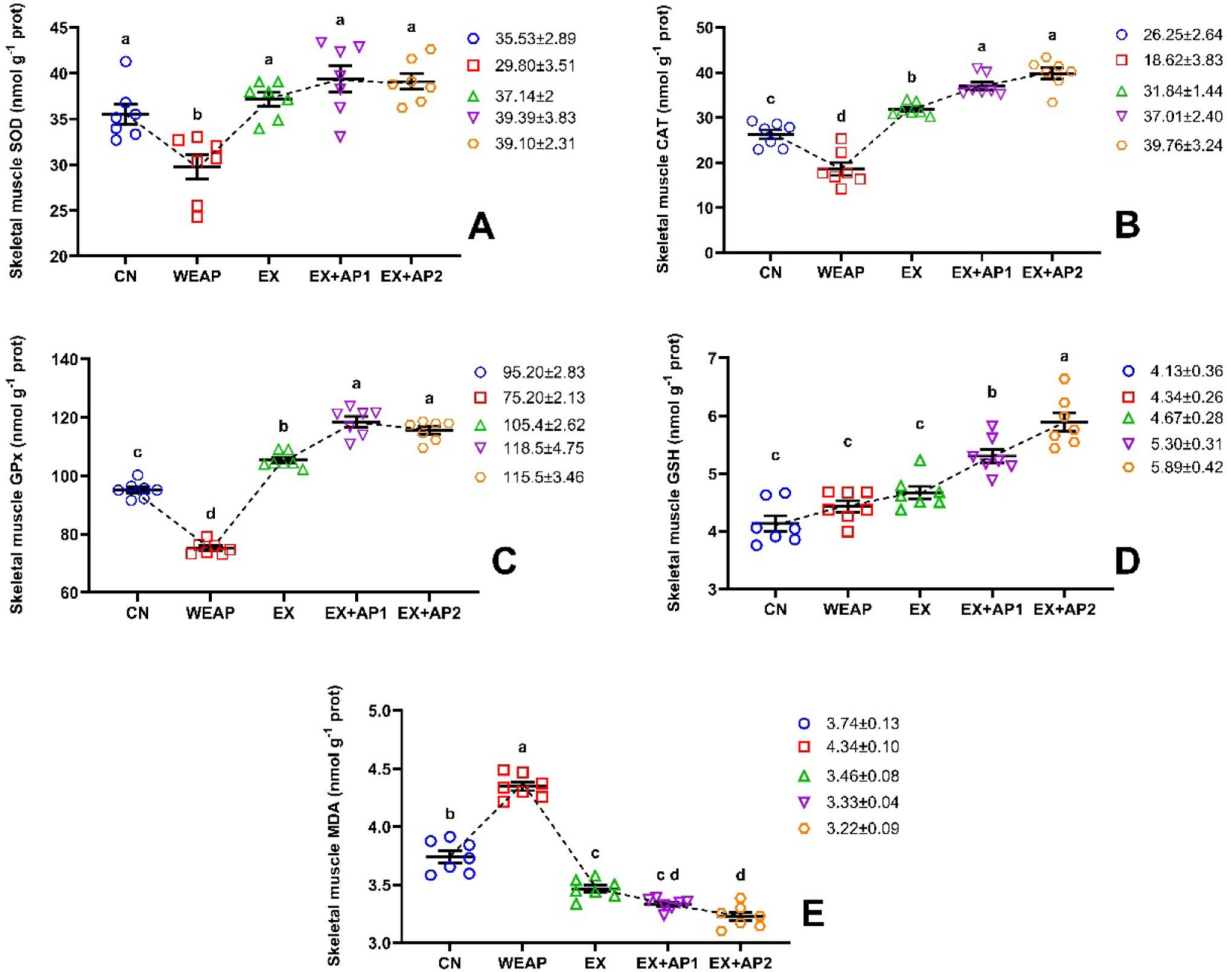
Oxidative stress and antioxidant parameters in skeletal muscle. **A** Superoxide dismutase (SOD), **B** catalase (CAT), **C** glutathione peroxidase (GSH-Px), **D** reduced glutathione (GSH), and **E** malondialdehyde (MDA). Data are presented as mean ± SEM. One-way ANOVA followed by Bonferroni post hoc test; bars not sharing a common letter differ significantly (p < 0.05). *CN* Control, *WEAP* Without exercise and apilarnil, *EX* Exercise, *EX* + *AP1* Exercise + apilarnil (0.2 g^.^kg^−1^), *EX* + *AP2* Exercise + apilarnil (0.4 g^.^kg^−1^)

Direct comparisons with EX indicated selective effects of apilarnil supplementation. Both EX + AP1 and EX + AP2 showed significantly higher CAT and GSH-Px activities than EX (p < 0.05), while SOD activity did not differ significantly (p > 0.05). GSH levels were higher in EX + AP1 compared with EX (p < 0.05), with a further increase observed in EX + AP2 (p < 0.05 vs EX + AP1). For lipid peroxidation, EX + AP2 exhibited significantly lower MDA levels than EX (p < 0.05), whereas EX + AP1 did not differ significantly from EX (p > 0.05) (Fig. 3A–E).

### Oxidative stress and antioxidant parameters in cardiac muscle

Relative to CN, the WEAP group showed significantly lower antioxidant enzyme activities (SOD, CAT, and GSH-Px) (p < 0.05), while MDA levels were significantly higher (p < 0.05). GSH levels did not differ significantly between WEAP and CN (p > 0.05).

Compared with WEAP, the EX group showed significantly higher SOD, CAT, and GSH-Px activities (p < 0.05). MDA levels were significantly lower in EX than WEAP (p < 0.05), whereas GSH levels remained statistically unchanged (p > 0.05).

**Fig. 4:**
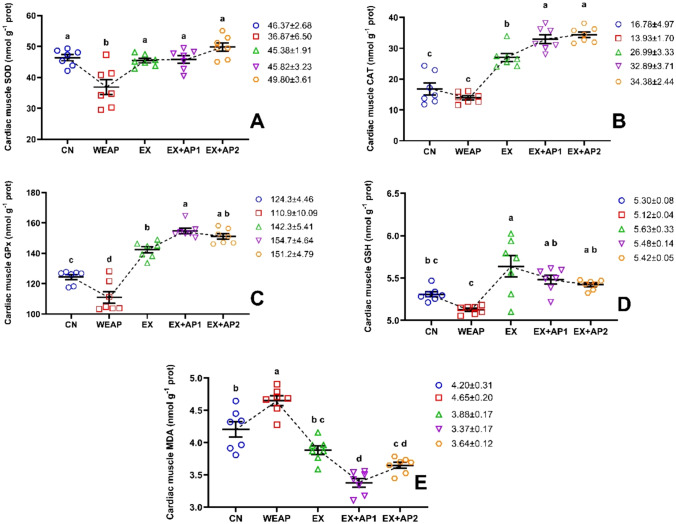
Oxidative stress and antioxidant parameters in cardiac muscle. **A** Superoxide dismutase (SOD), **B** catalase (CAT), **C** glutathione peroxidase (GSH-Px), **D** reduced glutathione (GSH), and **E** malondialdehyde (MDA). Data are presented as mean ± SEM. One-way ANOVA followed by Bonferroni post hoc test; bars not sharing a common letter differ significantly (p < 0.05). *CN* Control, *WEAP* Without exercise and apilarnil, *EX* Exercise, *EX* + *AP1* Exercise + apilarnil (0.2 g^.^kg^−1^), *EX* + *AP2* Exercise + apilarnil (0.4 g^.^kg^−1^)

Direct comparison with EX indicated that both EX + AP1 and EX + AP2 exhibited significantly higher CAT and GSH-Px activities (p < 0.05), while SOD activity did not differ significantly (p > 0.05). GSH levels were higher in EX + AP1 compared with EX (p < 0.05), with a further increase in EX + AP2 (p < 0.05 vs EX + AP1). For lipid peroxidation, EX + AP2 showed significantly lower MDA levels than EX (p < 0.05), whereas EX + AP1 did not differ significantly from EX (p > 0.05) (Fig. 4A–E).

### Swimming performance

Compared with WEAP, the EX group showed a significantly longer swimming duration (WEAP: 126.86 min vs EX: 152.29 min, p < 0.05).

**Fig. 5:**
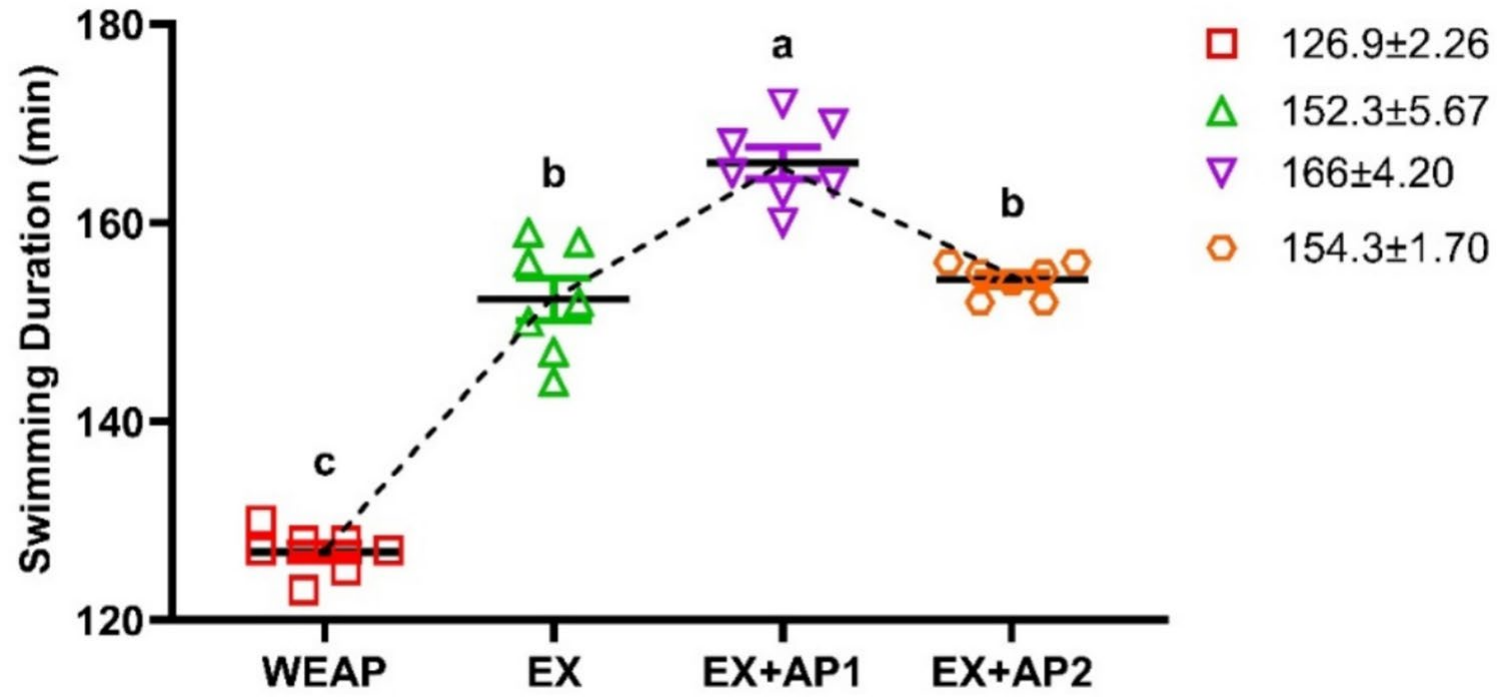
Swimming performance. Data are presented as mean ± SEM. One-way ANOVA followed by Bonferroni post hoc test; bars not sharing a common letter differ significantly (p < 0.05). *CN* Control, *WEAP* Without exercise and apilarnil, *EX* Exercise, *EX* + *AP1* Exercise + apilarnil (0.2 g^.^kg^−1^), *EX* + *AP2* Exercise + apilarnil (0.4 g^.^kg^−1^)

Compared directly with EX, swimming duration was significantly higher in EX + AP1 (166.00 min; p < 0.05), whereas EX + AP2 (154.29 min) did not differ significantly from EX (p > 0.05) and was significantly lower than EX + AP1 (p < 0.05) (Fig. 5).

## Discussion

This study investigated the effects of apilarnil supplementation at two different doses (0.2 and 0.4 g^.^kg^−1^) on redox homeostasis, inflammatory response, and endurance performance in skeletal and cardiac muscle tissues under exercise-induced physiological stress conditions. Apilarnil, a drone-larvae-derived bee product, contains proteins, essential amino acids, lipids, sterols, vitamins, and phenolic compounds that may collectively influence oxidative stress regulation, inflammatory signaling, and tissue-repair processes (Sidor and Dżugan 2020; Moraru et al. 2024a; Çakar and Şimşek 2025). Unlike bee pollen and propolis, which primarily exert antioxidant or immunomodulatory effects through plant-derived polyphenols (Denisow and Denisow‐Pietrzyk 2016; Mărgăoan et al. 2019; El-Seedi et al. 2022; Woźniak et al. 2022), apilarnil includes animal-derived macromolecules and sterol-related compounds that may be more directly associated with metabolic recovery mechanisms following exercise (Mărgăoan et al. 2017; Sidor and Dżugan 2020; Çakar et al. 2025).

Given the antioxidant and anti-inflammatory properties attributed to apilarnil in the literature (Yücel et al. 2019; Moraru et al. 2024a; Çakar and Şimşek 2025), the swimming exercise protocol used in this study was designed as a controlled physiological stress model to evaluate the biological activity of this bee-derived compound. Comparisons between EX-EX + AP1 and EX-EX + AP2 under standardized exercise conditions enabled assessment of the dose-dependent effects of apilarnil, while the WEAP-EX comparison served as a reference for exercise-induced redox and inflammatory responses. This design allowed differentiation between training-related physiological adaptations and apilarnil-related effects.

Exercise-induced oxidative stress and inflammatory responses have been extensively described in the literature. During intense or exhaustive exercise, increased oxygen consumption leads to elevated production of ROS, which can accelerate lipid peroxidation, increase cellular damage markers, and trigger cytokine-mediated inflammatory responses (Wang et al. 2021; Thirupathi et al. 2021b; Duranti 2023). However, ROS generated during exercise also act as signaling molecules that regulate physiological adaptation by stimulating endogenous antioxidant defense systems through redox-sensitive pathways (Powers et al. 2016, 2022; Margaritelis et al. 2020). In this context, the WEAP-EX comparison in the present study reflects the training-related effects of the swimming protocol. The observed alterations in oxidative stress–antioxidant balance and inflammatory markers in skeletal and cardiac muscle tissues are consistent with previous evidence indicating that exercise-induced redox processes influence both local and systemic physiological response (Margaritelis et al. 2020; Taherkhani et al. 2021; Powers et al. 2022).

When the intervention-attributable contribution of apilarnil under training conditions was evaluated, the apilarnil-supplemented groups exhibited lower levels of the lipid peroxidation marker MDA compared with the EX group. Similarly, proinflammatory cytokines (TNF-α, IL-1β, and IL-6) were suppressed, while antioxidant defense indicators, including SOD, CAT, and GSH-Px activities as well as GSH levels, shifted toward a more favorable profile. These findings suggest that apilarnil supplementation may modulate exercise-induced oxidative stress and inflammatory responses. This observation is biologically consistent with the composition of apilarnil as a bee-derived product rich in bioactive compounds, particularly phenolic acids and flavonoids (Silici 2019; Yücel et al. 2019; Moraru et al. 2024b), and with experimental studies reporting protective antioxidant and anti-inflammatory effects of apilarnil in lipopolysaccharide-induced inflammatory damage models (Doğanyiğit et al. 2020; Inandiklioglu et al. 2021; El-Seedi et al. 2022). In addition, recent analytical studies describing the phenolic profile of apilarnil, together with review articles highlighting its functional food and nutraceutical potential, provide a theoretical framework supporting the role of apilarnil in modulating redox homeostasis and inflammatory responses (Silici 2019; Sidor and Dżugan 2020; İvgin Tunca and Arslan 2024; Moraru et al. 2024b). Consistent with this theoretical framework, our findings indicate that apilarnil supplementation improved the redox and inflammatory profile of exercise-exposed tissues. However, the specific bioactive components responsible for these effects and the underlying molecular mechanisms were not directly investigated in the present study. Therefore, the observed biochemical modulation should be interpreted in the context of previously reported antioxidant and anti-inflammatory mechanisms associated with apilarnil and requires further mechanistic investigation (Doğanyiğit et al. 2020; Inandiklioglu et al. 2021; El-Seedi et al. 2022).

One of the most notable findings of the present study is the absence of a linear relationship between biochemical responses and performance outcomes. Improvements observed in biochemical markers did not translate into proportional increases in functional performance. The time to exhaustion during the exhaustive swimming test was highest in the EX + AP1 group, whereas performance in the EX + AP2 group remained close to that of the EX group and lower than that of EX + AP1. These results suggest that improvements in redox balance and inflammatory markers should not be interpreted as directly and unidirectionally determining performance outcomes (Powers et al. 2016; Mason et al. 2020). Although the EX + AP2 group exhibited a biochemical profile that was comparable to, or in some parameters more favorable than, that of the EX + AP1 group, this did not result in superior performance. Therefore, the present dataset does not support a simplified causal model in which “biomarker improvement equals performance enhancement.” Instead, the findings indicate a potential dissociation between biochemical adaptations and functional outcomes, particularly across different supplementation doses (Braakhuis and Hopkins 2015; Margaritelis et al. 2020).

This type of “biochemistry-performance dissociation” has been frequently emphasized in the literature on antioxidant and nutraceutical supplementation in endurance exercise (Braakhuis and Hopkins 2015; Mason et al. 2020). Although antioxidant supplementation can reduce oxidative stress and inflammatory markers, its effects on endurance performance are often limited and context-dependent (Mason et al. 2020). Moreover, exercise-induced ROS function not only as damaging agents but also as signaling molecules involved in physiological adaptation, suggesting that excessive antioxidant modulation may interfere with adaptive responses (Powers et al. 2016). These observations help explain the dose–response pattern observed in the present study. While apilarnil supplementation improved redox balance and inflammatory markers, performance outcomes suggested the presence of an optimal dose range. Low-dose apilarnil (AP1) produced a clearer improvement in endurance performance, whereas the higher dose (AP2) resulted in favorable biochemical changes without comparable functional gains. This pattern indicates that endurance performance is influenced not only by redox and inflammatory status but also by multiple interacting physiological factors (Margaritelis et al. 2020; Powers et al. 2022).

In the present study, structural and functional parameters directly reflecting training adaptation-such as muscle mass, hypertrophy, histomorphometric characteristics, mitochondrial biogenesis markers, muscle glycogen content, and other indicators of exercise adaptation-were not measured. Therefore, the interpretation of performance differences observed among the EX, EX + AP1, and EX + AP2 groups in terms of changes in muscle mass or adaptation level is limited by the scope of the available dataset. This limitation is consistent with previous reports indicating that biochemical markers, such as antioxidant enzyme activities and inflammatory cytokine levels, do not always show a linear relationship with performance outcomes (Powers et al. 2016; Mason et al. 2020). Accordingly, the lack of parallel changes between biochemical improvements and endurance performance in the present study suggests that this dissociation cannot be explained solely by redox balance and inflammatory responses. Rather, it may reflect physiological mechanisms related to energy metabolism and muscle adaptation that were not directly assessed in the current experimental design (Doğanyiğit et al. 2020; Inandiklioglu et al. 2021; Çakar et al. 2025).

### Limitations of the study

This study has several methodological limitations. First, the experimental period was limited to 14 days, preventing evaluation of the long-term effects of apilarnil supplementation on exercise adaptation. In addition, endurance performance was assessed only by time to exhaustion in the swimming test, and multidimensional performance indicators were not included. Second, structural markers of muscle adaptation-such as muscle mass, fiber-type distribution, mitochondrial biogenesis markers, and muscle glycogen content-were not measured, limiting interpretation of the relationship between biochemical responses and performance outcomes. Third, although apilarnil contains multiple bioactive compounds, the specific components responsible for the observed effects and the underlying molecular mechanisms were not directly investigated. Furthermore, biochemical measurements were performed only immediately after the exhaustive exercise test, preventing evaluation of potential time-dependent responses. Another limitation is that only male rats were included to minimize hormonal variability associated with the estrous cycle. Finally, as the study was conducted in an animal model, the generalizability of the findings to other exercise models or human populations should be interpreted with caution.

## Conclusion

In this study, the effects of apilarnil supplementation on redox balance, inflammatory response, and endurance performance in skeletal and cardiac muscle tissues under exercise-induced physiological stress were evaluated using an experimental model. The findings indicate that the swimming exercise protocol induced significant changes in oxidative stress and inflammatory markers, while apilarnil supplementation exerted a modulatory effect on these processes. The reduction in lipid peroxidation and proinflammatory cytokine levels, together with improvements in antioxidant defense indicators in the apilarnil-supplemented groups, suggests that apilarnil may regulate exercise-induced redox and inflammatory responses at the biochemical level.

From a functional perspective, endurance performance was more pronounced in the low-dose apilarnil group, whereas higher-dose supplementation did not produce comparable performance gains despite favorable biochemical changes. This finding indicates that changes in biochemical markers do not necessarily translate into proportional improvements in performance outcomes and suggests that the effects of apilarnil under exercise conditions may follow a dose-sensitive pattern.

Overall, apilarnil appears to be a potential nutraceutical capable of modulating oxidative stress and inflammatory responses in muscle tissues during exercise loading; however, its influence on performance outcomes likely reflects a multifactorial process that cannot be explained solely by biochemical markers. Further studies are needed to clarify the mechanisms underlying these effects and the optimal supplementation conditions.

## Data Availability

The datasets generated and/or analyzed during the current study are available from the corresponding author upon reasonable request.

## Abbreviations

AP: Apilarnil
CN: Control group
WEAP: Without Exercise and Apilarnil group
EX: Exercise group
EX + AP1: Exercise + Apilarnil (0.2 g·kg⁻1) group
EX + AP2: Exercise + Apilarnil (0.4 g·kg⁻1) group
ROS: Reactive Oxygen Species
MDA: Malondialdehyde
GSH: Reduced Glutathione
SOD: Superoxide Dismutase
CAT: Catalase
GSH-Px: Glutathione Peroxidase
CK: Creatine Kinase
CK-MB: Creatine Kinase-MB isoenzyme
LDH: Lactate Dehydrogenase
TNF-α: Tumor Necrosis Factor-alpha
IL-1β: Interleukin-1 beta
IL-6: Interleukin-6
PBS: Phosphate-Buffered Saline
BSA: Bovine Serum Albumin
DTN: 5,5′-Dithiobis-(2-nitrobenzoic acid)
TCA: Trichloroacetic Acid
NBT: Nitroblue Tetrazolium
NF-κB: Nuclear Factor kappa-light-chain-enhancer of activated B cells
Nrf2: Nuclear factor erythroid 2–related factor 2
